# Sinapic Acid Ameliorates Doxorubicin-Induced Cardiotoxicity in H9c2 Cardiomyoblasts by Inhibiting Oxidative Stress Through Activation of the Nrf2 Signaling Pathway

**DOI:** 10.3390/antiox14030337

**Published:** 2025-03-13

**Authors:** Tsendsuren Tungalag, Hyung-Sub Kang, Dong Kwon Yang

**Affiliations:** 1 Department of Veterinary Pharmacology and Toxicology, College of Veterinary Medicine, Jeonbuk National University, Iksan 54596, Jeollabuk-do, Republic of Korea; mgljuuh@jbnu.ac.kr; 2 Biosafety Research Institute and College of Veterinary Medicine, Jeonbuk National University, Iksan 54596, Jeollabuk-do, Republic of Korea

**Keywords:** sinapic acid, oxidative stress, reactive oxygen species, ER stress, Nrf2

## Abstract

The use of doxorubicin (Dox) is restricted because of its cardiotoxicity, which poses a significant mortality risk for cancer patients, despite being a highly effective antibiotic for treating various types of cancer. Therefore, identifying substances or developing preventive strategies against Dox-induced cardiotoxicity is crucial. This study was conducted to determine whether sinapic acid (SA), a phenolic compound with a range of pharmacological effects, could protect against Dox-induced cardiotoxicity in H9c2 cardiomyoblasts. To investigate the preventive effect of SA, H9c2 cardiomyoblasts treated with Dox were pretreated with SA at various concentrations. SA effectively rescued the cells from Dox-induced cardiotoxicity. Additionally, SA significantly reduced oxidative stress by inhibiting mitochondrial dysfunction and endoplasmic reticulum stress. SA also suppressed the expression of MAPK proteins. As for the underlying mechanism of SA’s protective effect against Dox-induced cardiotoxicity, SA activated nuclear factor erythroid-2-related factor (Nrf2) by facilitating its movement from the cytosol to the nucleus and increasing the expression of its target antioxidative genes. In summary, this study demonstrated that SA protects H9c2 cardiomyoblasts from Dox-induced cardiotoxicity by inhibiting oxidative stress by the activation of Nrf2-related signaling pathway. Our findings enhance the development of therapeutic strategies to mitigate cardiac toxicity caused by Dox, highlighting the potential antioxidant effect of SA in Dox-treated H9c2 cardiomyoblasts.

## 1. Introduction

Doxorubicin (Dox), also known as adriamycin or anthracycline, is a highly potent antineoplastic antibiotic widely used to treat various cancers, including hematologic malignancies and prostate cancer [1]. Dox is also associated with long-term side effects such as gonadal toxicity, nephrotoxicity, and cardiotoxicity [2]. Notably, Dox treatment can lead to dilated cardiomyopathy, which may progress to heart failure [3]. Depending on the timing of onset, Dox-induced cardiotoxicity can be classified into three types: acute onset, occurring within two weeks of treatment completion; chronic onset divided into early-onset, arising within a year; and late-onset, which can develop years later, even up to 10 years after treatment [4]. Dox causes pathological changes in heart cells, including DNA damage, senescence, telomere shortening, apoptosis, reduced migratory capacity, cell cycle arrest, and increased reactive oxygen species (ROS) production [5]. Specifically, H9c2 cardiomyoblasts treated with low concentrations of Dox exhibit minor alterations while maintaining cell membrane integrity, such as changes in structural proteins and activation of pro-apoptotic proteins. In contrast, treatment with higher concentrations of Dox induces severe morphological changes, including nuclear and mitochondrial damages and cytoplasmic vacuolization [6]. Consequently, significant efforts have been made to prevent Dox-induced cardiac toxicity. One such approach involves the development of encapsulated anthracyclines, which have been shown to be effective with reduced cardiotoxic effects [7]. Additionally, new Dox analogs, such as aldoxorubicin, camsirubicin, and annamycin, have been developed and used to treat cancers while limiting cardiac toxicity [8]. However, clinical data on their safety profiles remain inconclusive [9]. Therefore, it is essential to identify new compounds that can prevent Dox-induced cardiotoxicity.

The phenolic compound sinapic acid (SA), which occurs naturally in free, ester, or glycoside forms, is found in many plants, including fruits, vegetables, cereals, some spices, oranges, and oil seeds [10]. SA possess the beneficial effects, including anxiolytic, vasodilatory, anti-inflammatory, and antioxidant properties [11]. Notably, SA exhibits potential antioxidative effects by restoring endogenous antioxidants such as SOD, GST, catalase, and GPx [12]. Previous studies have shown that SA provides beneficial effects against various diseases, including Alzheimer’s disease, alcoholic liver disease [13], obesity, Parkinson’s disease, and cardiac diseases, primarily through the inhibition of oxidative stress [14].

Oxidative stress, which disrupts the balance between the ROS production and the endogenous antioxidative system, is a major cause of Dox-induced cardiotoxicity [15]. Dox easily accumulates within mitochondria, further disrupting the redox cycle at the electron transport chain by impairing mitochondrial complex protein functions [16]. This disruption leads to a large accumulation of ROS, including the production of highly reactive and toxic hydroxyl radicals, which cause further mitochondrial dysfunction and increase ROS generation [17]. Therefore, addressing oxidative stress has become a key focus in efforts to suppress Dox-induced cardiotoxicity.

Previous research reported that SA protects the heart from Dox-induced cardiotoxicity by inhibiting oxidative stress, inflammation, and apoptosis in rat models [18]. In particular, the level of endogenous oxidative stress inducer was increased, while the levels of antioxidants were decreased in Dox-treated rats, whereas these changes were dramatically inhibited by SA pretreatment [18]. Another study also demonstrated that SA has protective effects against cardiomyopathy by inhibiting oxidative stress, inflammation, and apoptosis through the NRF2/HO-1 and NF-κB pathway in streptozocin-induced diabetic rats [19]. These findings suggest that oxidative stress is a critical factor in Dox-induced cardiotoxicity.

Therefore, this study aimed to examine the beneficial effects of SA on Dox-induced cardiotoxicity, specifically by investigating the mechanisms related to the induction of oxidative stress in H9c2 cardiomyoblasts. Furthermore, it sought to clarify the relationship between the oxidative stress-inhibiting effects of SA and the NRf2-related signaling mechanisms, which are crucial for suppressing oxidative stress.

## 2. Materials and Methods

### 2.1. SA and Dox Treatment in Cell Culture

H9c2 cardiomyoblasts purchased from the KCLB (Seoul, Republic of Korea) were cultured in DMEM containing 10% FBS (GIBCO-BRL, Grand Island, NE, USA) and 1% antibiotics and maintained at 37 °C with 5% CO_2_. After dissolving SA and Dox in dimethyl sulfoxide (DMSO, Sigma Aldrich Co., St. Louis, MO, USA), the cells were pretreated with 100, 200, or 400 µM SA for 24 h; they were then treated with 5 µM Dox for an additional 24 h.

### 2.2. Cell Viability Assay

A 0.5 mg/mL MTT (Sigma) was added to cells treated with SA and Dox. After incubation at 37 °C for 2 h, DMSO was added to dissolve the formazan and measured the absorbance was at 570 nm using a spectrophotometer (SpectraMax M5; Molecular Devices, Sunnyvale, CA, USA).

### 2.3. Hoechst 33342 Dye Staining

Nuclear staining using Hoechst 33342 dye (ThermoFisher Scientific Inc., Waltham, MA, USA) was performed to detect apoptotic cells. After fixed with 4% paraformaldehyde (Sigma), the cells were incubated with 500 ng/mL Hoechst 33342 dye at 37 °C for 30 min. The nuclei were observed using a fluorescence microscope (Olympus Corp., Tokyo, Japan). The apoptotic index was calculated as the ratio of apoptotic cells to the total cells.

### 2.4. Measurement of Intracellular ROS

The level of ROS was measured using DCFH-DA dye (ThermoFisher). Cells were incubated with 1 µM DCFH-DA dye for 30 min at 37 °C. The cells were then observed using a fluorescence microscope (Olympus).

### 2.5. Analysis of Mitochondrial Membrane Potential (MMP) and Mitochondrial ROS Production

MMP immunostaining was assessed using JC-1 reagent (ThermoFisher), and mitochondrial ROS production was measured using the MitoSOX Assay Kit (ThermoFisher). For JC-1 immunostaining, the cells were incubated with 5 μg/mL JC-1 dye for 20 min at 37 °C and observed using a fluorescence microscope (Olympus). Green fluorescence (indicating depolarized mitochondria) was detected at an 515/529 nm (excitation/emission wavelength), while red fluorescence (indicating polarized mitochondria) was detected at 485/590 nm (excitation/emission wavelength). Mitochondrial ROS was detected using the MitoSOX Red Mitochondrial Superoxide Indicator. Cells were incubated with 5 µM MitoSOX reagent for 10 min at 37 °C. Images were captured using a live-cell imaging system (Oxford Instruments, Oxfordshire, UK).

### 2.6. Western Blot Analysis

Proteins were extracted using RIPA (LPS Solution, Daejeon, Republic of Korea) with phosphatase and protease inhibitors (Roche Diagnostics, Manheim, Germany). The lysates were then centrifuged, and the supernatant was collected. Proteins were separated by SDS-PAGE and transferred onto PVDF membranes (Bio-Rad Laboratories, Hercules, CA, USA). The membranes were blocked with 5% BSA for 1 h at RT. After blocking and incubated with primary antibodies for 6 h at 4 °C, followed by incubation with HRP-conjugated secondary antibodies for 1 h at RT. Proteins were detected using an enhanced chemiluminescence (Millipore Inc., Billerica, MA, USA) and visualized using a Chem-iDoc imaging system (Cleaver Scientific Ltd., Warwickshire, UK). β-actin and Lamin β1 were used as loading controls for total protein and nuclear fractionation, respectively. The antibodies used in these experiments are listed in Supplementary Table S1.

### 2.7. Nuclear Fractionation of H9c2 Cardiomyoblasts

Nuclear fractionation was performed by using the Nuclear Extraction Kit (Abcam, Cambridge, UK). After lysis with hypotonic buffer with protease inhibitors, the cell pellet was collected by centrifugation at 3000× *g* for 10 min at 4 °C, and was then resuspended and incubated on ice for 30 min. Nuclei were collected following centrifugation at 24,000× *g* for 20 min at 4 °C.

### 2.8. Quantitative Real-Time Polymerase Chain Reaction (qRT-PCR)

Total RNA was extracted using the TRIzol reagent (Sigma). After synthesizing cDNA with the GoScriptTM Reverse Transcription System Kit (Promega, Madison, WI, USA), qRT-PCR was performed on a TaKaRa PCR machine (Takara Bio. Inc., Shiga, Japan) using SYBR qPCR Master Mix (Kapa Biosystems, Boston, MA, USA). Gene expression levels were normalized to 18S rRNA. The primer sequences are listed in Supplementary Table S2.

### 2.9. Transfection of Nrf2 Small Interfering RNA (Nrf2 siRNA)

Cells were transfected with either 30 nM Nrf2 siRNA or negative control siRNA (Santa Cruz Biotechnology, Dallas, TX, USA) using DharmaFECT transfection reagent (Horizon discovery Inc., Cambridge, UK). Following a 24-h incubation, the cells were pretreated with 400 µM SA for 24 h, then treated with 5 µM Dox for an additional 24 h.

### 2.10. Statistical Analysis

Statistical significance was established with a probability level of *p* < 0.05. The values are presented as the mean ± standard error of the mean (SEM). Data were analyzed using the Prism 5.02 software (GraphPad Software Inc., San Diego, CA, USA) with one-way analysis of variance (ANOVA) followed by Bonferroni post hoc tests.

## 3. Results

### 3.1. SA Prevents Dox-Induced Cytotoxicity in H9c2 Cardiomyoblasts

**Figure 1:**
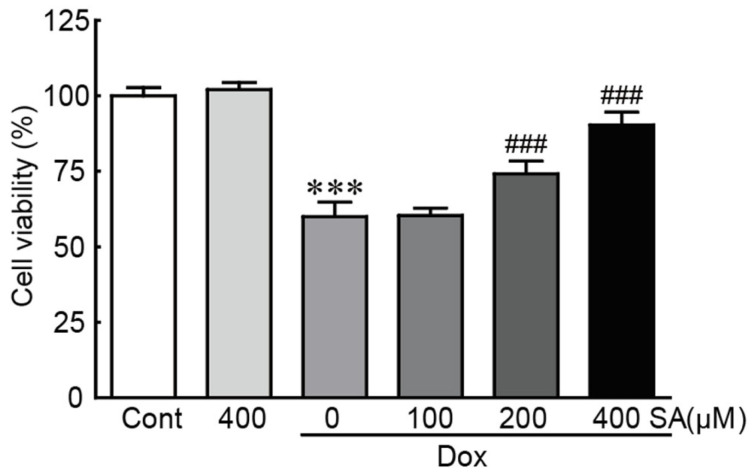
SA rescues H9c2 cardiomyoblasts from Dox-induced cardiotoxicity. Cell viability was assessed using an MTT assay (*n* = 3). *** *p* < 0.001 vs. control group. ### *p* < 0.001 vs. Dox-alone group. Dox, doxorubicin; Cont, control; SA, sinapic acid.

MTT assays were conducted to assess cell viability in H9c2 cardiomyoblasts treated with Dox, with or without SA pretreatment. Treatment with 400 μM SA alone did not induce cytotoxicity in H9c2 cardiomyoblasts. In contrast, cell viability was significantly reduced to 60.0% in cells treated with 5 μM Dox alone compared to control cells (Figure 1). However, the survival rate of cells pretreated with SA increased dose-dependently (60.0%, 74.1%, and 90.3% in 100, 200, and 400 μM SA-pretreated cells, respectively; Figure 1). These results indicate that SA effectively protected H9c2 cardiomyoblasts from Dox-induced cardiotoxicity.

### 3.2. SA Prevents Dox-Induced Apoptotic Cell Death in H9c2 Cardiomyoblasts

**Figure 2:**
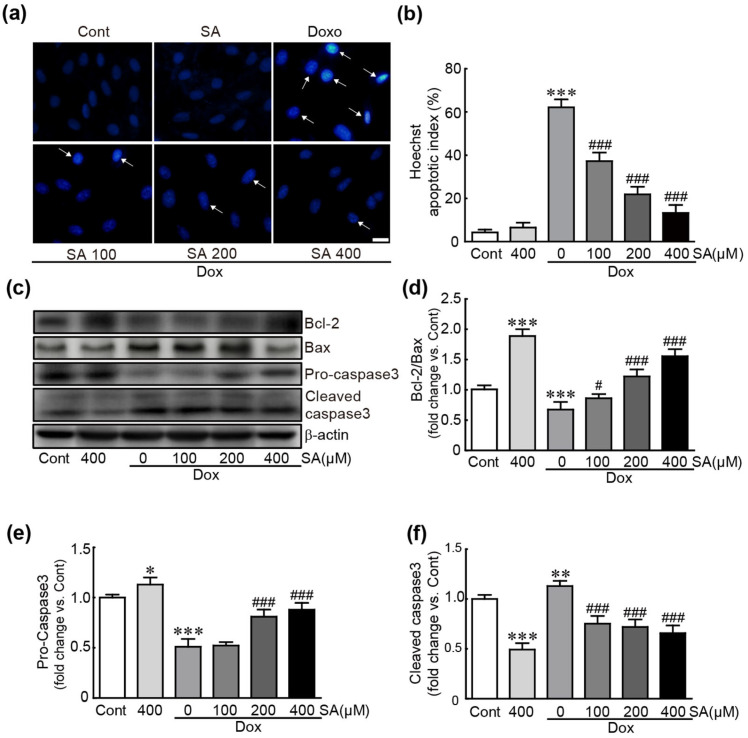
SA inhibits apoptotic cell death in Dox-induced cardiotoxicity. (**a**) Hoechst staining was used for nuclear visualization. (**b**) The apoptotic index is shown as the percentage of apoptotic cells relative to total cells, with counts performed in 10 fields (at least 30 cells per field). (**c**–**f**) Western blot analysis of apoptosis-related proteins. * *p* < 0.05, ** *p* < 0.01, and *** *p* < 0.001 vs. control group. # *p* < 0.05 and ### *p* < 0.001 vs. Dox-alone group. Arrow: apoptotic cells with nuclear DNA condensation. Dox, doxorubicin; Cont, control; SA, sinapic acid. Scale bar, 100 μm.

Hoechst 33342 staining and Western blot analysis of several apoptosis-related proteins were performed to determine whether SA could prevent apoptotic cell death in Dox-treated cells. Hoechst 33342 staining, a nuclear indicator, revealed that the number of apoptotic cells with nuclear condensation significantly increased to 62.13% in cells treated with Dox alone. In contrast, in SA-pretreated cells, the proportion of apoptotic cells decreased as the concentration increased (37.2%, 21.9%, and 13.3% in 100, 200, and 400 μM SA-pretreated cells, respectively; Figure 2a,b). Additionally, Western blot analysis revealed that Bcl-2, an anti-apoptotic protein, significantly reduced, whereas Bax, a pro-apoptotic protein, significantly increased in cells treated with Dox alone (0.6-fold change in Bcl-2/Bax ratio vs. control cells; Figure 2c,d). However, in SA-pretreated cells, their expression restored (0.9-, 1.2-, and 1.5-fold changes in Bcl-2/Bax ratio for 100, 200, and 400 μM SA-pretreated cells, respectively; Figure 2c,d).

Moreover, caspase-3, a pro-apoptotic protein, was activated in Dox-treated cells, as evidenced by a decrease in pro-caspase-3 (the inactive form) and an increase in cleaved-caspase-3 (the active form) (0.5- and 1.1-fold changes in pro- and cleaved-caspase-3, respectively, compared to control cells; Figure 2c,e,f). Notably, in SA-pretreated cells, pro-caspase-3 levels increased (0.52-, 0.81-, and 0.88-fold changes in 100, 200, and 400 μM SA-pretreated cells, respectively), while cleaved-caspase-3 levels decreased (0.8-, 0.7-, and 0.7-fold changes in 100, 200, and 400 μM SA-pretreated cells, respectively; Figure 2c,e,f). These results suggest that SA effectively preserves cells from Dox-caused apoptotic responses.

### 3.3. SA Protects H9c2 Cardiomyoblasts Against Dox-Induced Oxidative Stress

**Figure 3:**
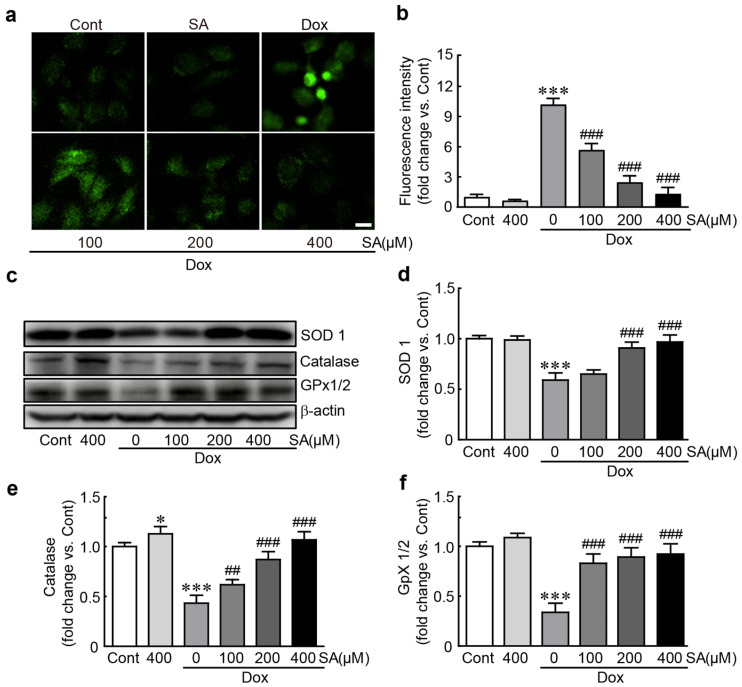
SA inhibits oxidative stress in Dox-induced cardiotoxicity. (**a**,**b**) Green fluorescence intensity was measured using DCFH-DA staining. (**c**–**f**) Western blot analysis of antioxidants (*n* = 5). * *p* < 0.05 and *** *p* < 0.001 vs. control group. ## *p* < 0.01 and ### *p* < 0.001 vs. Dox-alone group. Dox, doxorubicin; GPx, glutathione peroxidase; Cont, control; SA, sinapic acid. Scale bar, 100 μm.

DCFDA dye, a ROS indicator, was used to determine whether SA could inhibit ROS production in Dox-treated H9c2 cardiomyoblasts. DCFDA-positive cells significantly increased to 84.0% in Dox-treated cells, whereas it was dramatically reduced when SA was pretreated (46.8%, 20.3%, and 10.8% in 100, 200, and 400 μM SA-pretreated cells, respectively; Figure 3a,b). Additionally, Western blot analysis was conducted to measure the levels of antioxidants such as SOD1, catalase, and GPx1/2. In Dox alone-treated cells, the expression levels of these proteins decreased significantly (0.59-, 0.49-, and 0.34-fold changes for SOD1, catalase, and GPx1/2, respectively, compared to control cells; Figure 3c–f). However, when pretreated with SA, these antioxidants exhibited a dose-dependent increase (0.97-, 1.07-, and 0.92-fold changes vs. control cells, respectively; Figure 3c–f). Taken together, these results demonstrate that SA has potent antioxidant properties by inhibiting ROS production and enhancing the expression of endogenous antioxidants in Dox-treated cells. protected H9c2 cardiomyoblasts from Dox-induced cardiotoxicity.

### 3.4. SA Inhibits Dox-Induced Mitochondrial Oxidative Stress and Mitochondrial Dysfunction in H9c2 Cardiomyoblasts

**Figure 4:**
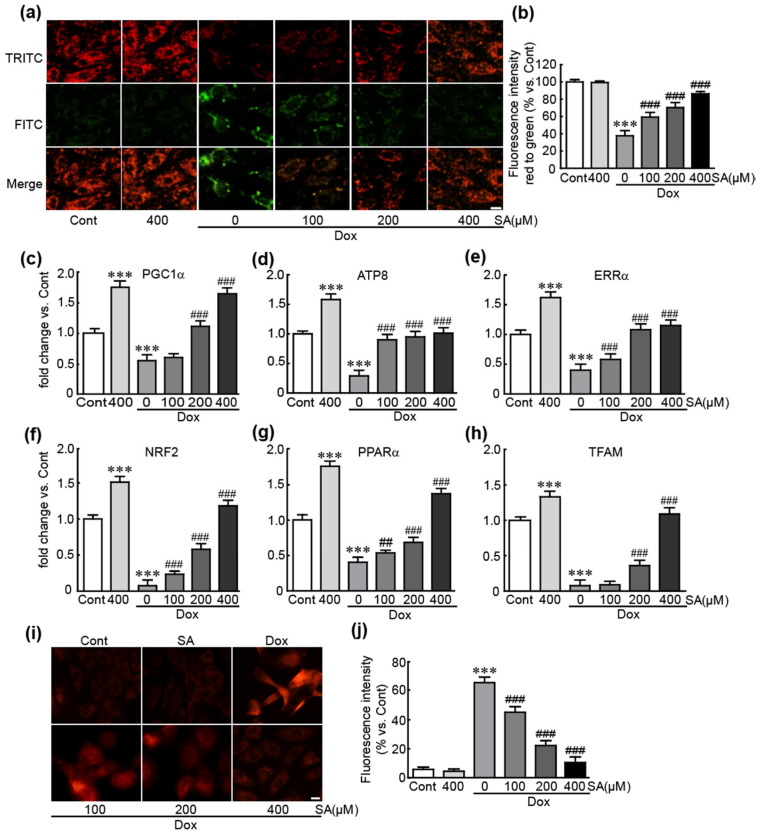
SA suppresses mitochondrial dysfunction and mitochondrial ROS production in Dox-induced cardiotoxicity. MMP was assessed by JC-1 dye staining. (**a**,**b**) The intensities of green and red fluorescence were measured using JC-1 staining. (**c**–**h**) Mitochondrial functional genes were analyzed by qRT-PCR in triplicate. MitoSox staining. (**i**,**j**) The intensity of red fluorescence was measured using MitoSOX staining. *** *p* < 0.001 vs. control group. ## *p* < 0.01 and ### *p* < 0.001 vs. Dox-alone group. Dox, doxorubicin; Cont, control; SA, sinapic acid. Scale bar, 100 μm.

To clarify the preventive effects of SA against Dox-induced mitochondrial dysfunction, JC-1 dye immunostaining and qRT-PCR analysis of various energy metabolism-related genes were performed on Dox-treated cells with or without SA pretreatment. JC-1 dye immunostaining revealed a predominance of green fluorescent cells, indicating a loss of MMP with a 37.7% reduction in the red/green fluorescence ratio compared to control cells in Dox-treated cells (Figure 4a,b). However, when pretreated with SA, red fluorescent cells, which indicate maintained MMP, gradually increased in a dose-dependent manner (59.3%, 70.3%, and 86.3% in the red/green fluorescence ratio vs. control cells, respectively; Figure 4a,b). Moreover, the mRNA levels of mitochondrial biogenesis-related genes, such as PGC1α, ATP8, ERRα, NRF2, PPARα, and TFAM, were significantly reduced in Dox-treated cells. However, when pretreated with SA, the expression of these genes increased in a dose-dependent manner (Figure 4c–h). Furthermore, MitoSox staining, used to detect mitochondrial superoxide, revealed an increase in red fluorescent cells, indicating superoxide production, in Dox-treated cells (65.5%). In contrast, cells pretreated with SA showed a dose-dependent decrease in red fluorescent cells (45.3%, 22.3%, and 10.5% in 100, 200, and 400 μM SA-treated cells vs. control cells, respectively; Figure 4i,j). Collectively, these results demonstrate that SA effectively inhibits Dox-induced mitochondrial dysfunction and superoxide production in H9c2 cardiomyoblasts.

### 3.5. SA Prevents Dox-Induced ER Stress in H9c2 Cardiomyoblasts

**Figure 5:**
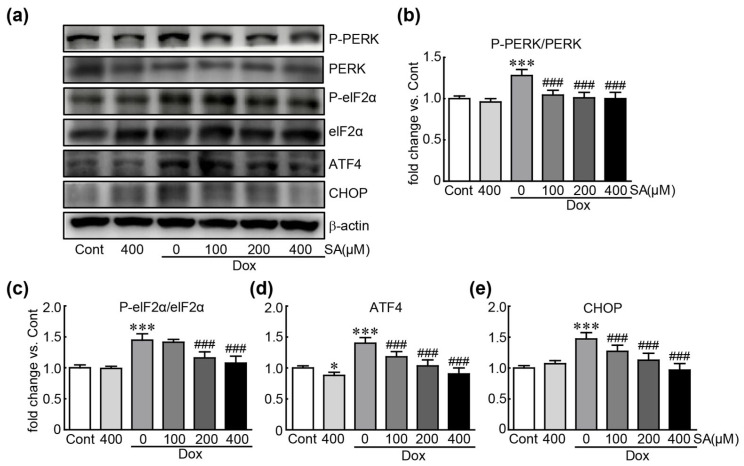
SA inhibits ER stress in Dox-induced cardiotoxicity. (**a**–**e**) Western blot analysis of ER stress-related proteins (*n* = 5). * *p* < 0.05 and *** *p* < 0.001 vs. control group. ### *p* < 0.001 vs. Dox-alone group. Dox, doxorubicin; ER stress, endoplasmic reticulum stress; Cont, control; SA, sinapic acid.

Western blot analysis was conducted to measure the expression levels of ER stress-related proteins, including PERK, eIF2α, ATF4, and CHOP, to clarify the inhibitory effects of SA on ER stress, which is closely linked to oxidative stress [20]. In cells treated with Dox alone, PERK and eIF2α were activated, as indicated by the increased levels of their phosphorylated forms, along with elevated levels of ATF4 and CHOP (1.2-, 1.5-, 1.4-, and 1.5-fold changes in p-PERK, p-eIF2α, ATF4, and CHOP, respectively, compared to control cells). However, these proteins were significantly reduced when SA were administered to Dox-treated cells (Figure 5). Notably, pretreatment with 400 μM SA inhibited the expression of these proteins to similar levels in control cells. These results indicate that SA exerts an inhibitory effect on Dox-induced ER stress in H9c2 cardiomyocytes.

### 3.6. SA Inhibits the MAPK Signaling Pathway in Dox-Treated H9c2 Cardiomyoblasts

**Figure 6:**
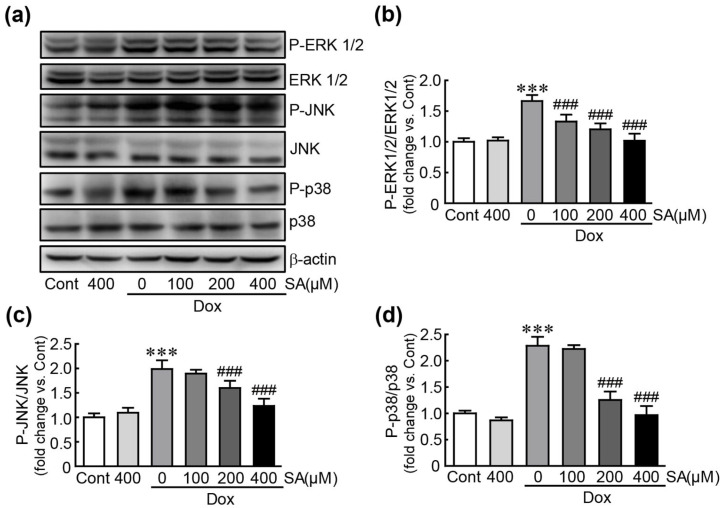
SA suppresses the MAPK signaling pathway in Dox-induced cardiotoxicity. (**a**–**d**) Western blot analysis of MAPK signaling-related proteins (*n* = 5). *** *p* < 0.001 vs. control group. ### *p* < 0.001 vs. Dox-alone group. Dox, doxorubicin; Cont, control; SA, sinapic acid.

Western blot analysis was performed to measure the expression levels of MAPK proteins, including ERK1/2, JNK, and p38. In cells treated with Dox alone, the phosphorylated forms of ERK, JNK, and p38 were significantly increased (1.7-, 2.3-, and 2.0-fold changes in p-ERK, JNK, and p38 vs. control cells, respectively; Figure 6). However, these phospho-proteins were significantly decreased following pretreatment with 100, 200, and 400 μM SA. Specifically, when pretreated with 400 μM SA reduced the activation of these proteins to similar levels in control cells (1.02-, 0.97-, and 1.23-fold changes in p-ERK, JNK, and p38, respectively, in 400 μM SA-pretreated cells vs. control cells; Figure 6). These results demonstrate that SA suppresses the Dox-induced activation of the MAPK signaling pathway.

### 3.7. SA Activates the Nrf2 Signaling Pathway in Dox-Treated H9c2 Cardiomyoblasts

**Figure 7:**
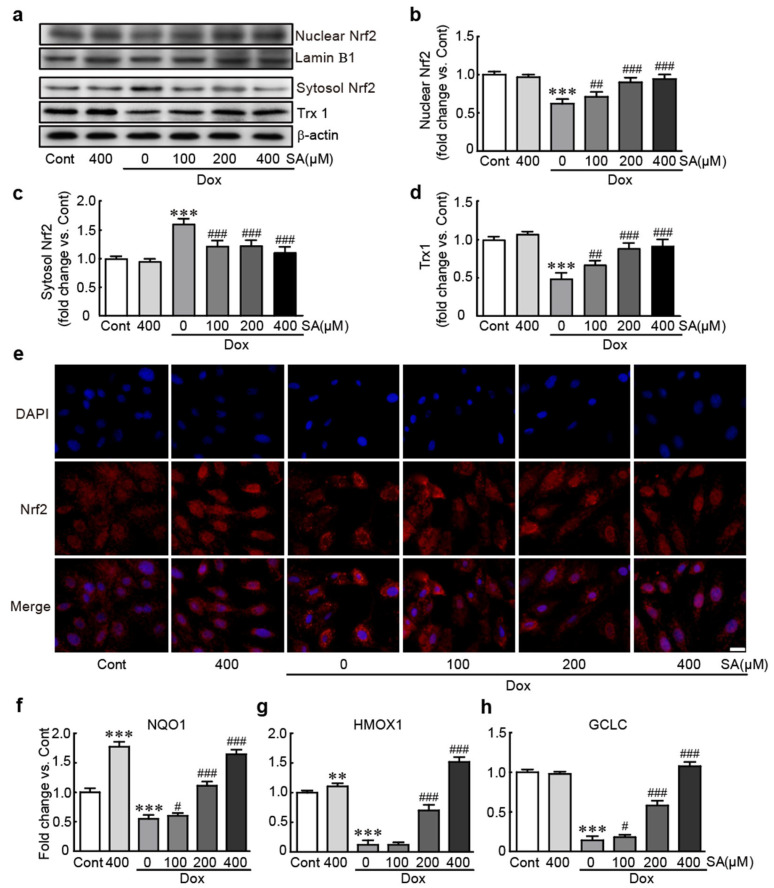
SA activates the Nrf2-related signaling pathway in Dox-induced cardiotoxicity. (**a**–**d**) Western blot analysis of nuclear and cytoplasmic Nrf2 and Trx1 (lamin B1 as a nuclear loading control and β-actin as a cytoplasmic loading control, *n* = 5). (**e**) Immunostaining of Nrf2 protein was performed to detect the localization of Nrf2 protein. (**f**–**h**) qRT-PCR analysis of Nrf2 target genes as oxidative stress-related genes. ** *p* < 0.01 and *** *p* < 0.001 vs. control group. # *p* < 0.05, ## *p* < 0.01 and ### *p* < 0.001 vs. Dox-alone group. Dox, doxorubicin; NQO1, NAD(P)H dehydrogenase (quinone); HMOX1, heme oxygenase 1; GCLC, glutamate–cysteine ligase, catalytic subunit. Cont, control; SA, sinapic acid. Scale bar, 100 μm.

We investigated the activation of the Nrf2 signaling pathway as a potential inhibitory mechanism of SA against Dox-induced cardiotoxicity. The results revealed that the expression level of nuclear Nrf2 protein, the active form, was significantly decreased in cells treated with Dox alone (0.62-fold change compared to control cells). However, pretreatment with SA caused to a significant increase in nuclear Nrf2 protein levels (0.71-, 0.9-, and 0.94-fold changes in cells pretreated with 100, 200, and 400 μM SA, respectively, compared to control cells; Figure 7a,b). Immunostaining of Nrf2 protein showed that the cells with nuclear-localized Nrf2 were increased when SA was pretreated (Figure 7e). Trx1, as a downstream protein of Nrf2 signaling pathway, was shown to decrease in Dox alone-treated cells and to increase in SA-pretreated cells (Figure 7a,d). Additionally, the mRNA expression of Nrf2 target genes, such as NQO1, HMOX1, and GCLC, was significantly reduced in Dox-treated cells (0.55-, 0.12-, and 0.14-fold changes in NQO1, HMOX1, and GCLC, respectively; Figure 7f–h). However, these mRNA levels increased in a dose-dependent manner following pretreatment with 100, 200, and 400 μM SA. In particular, pretreatment with 400 μM SA restored the expression of these genes to similar levels in control cells. Therefore, our findings suggest that SA protects cardiac cells through activating the Nrf2-related signaling pathway against the cardiotoxicity induced by Dox.

### 3.8. The Protective Effect of SA Is Abrogated in Nrf2-Silenced Dox-Treated H9c2 Cardiomyoblasts

**Figure 8:**
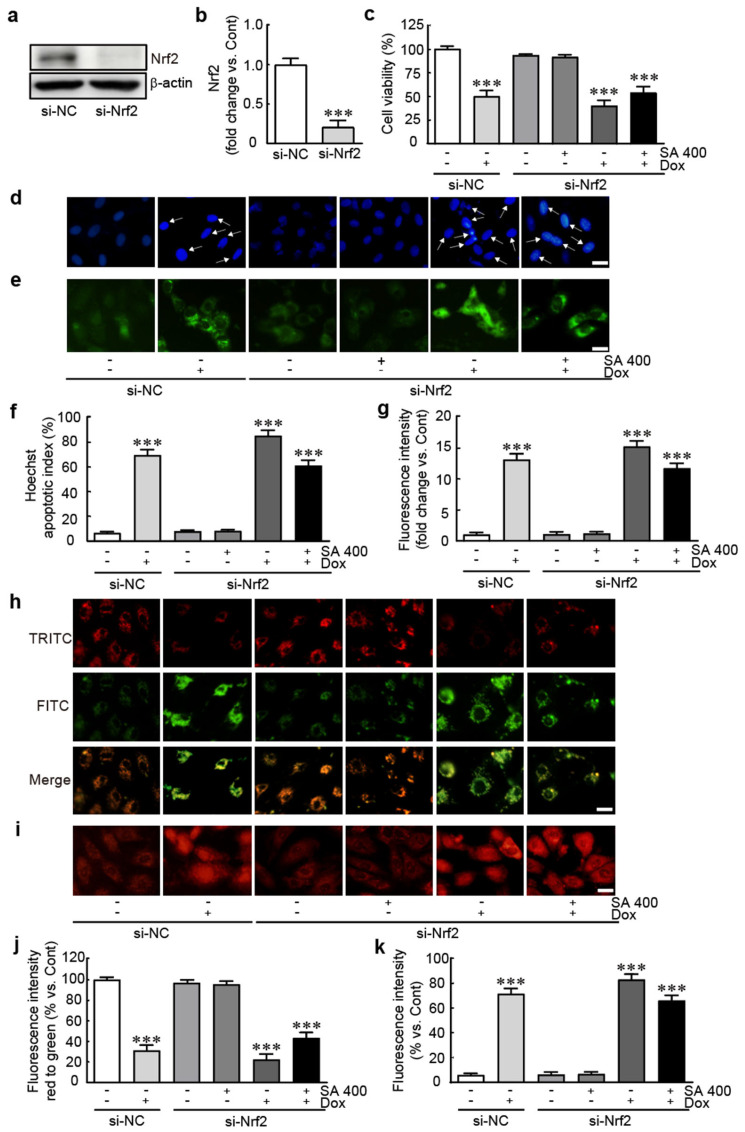
The protective effect of SA is abrogated in Nrf2-Silenced Dox-treated H9c2 cardiomyoblasts. (**a**,**b**) Western blot analysis of Nrf2 in NC siRNA or Nrf2 siRNA-transfected cells. (**c**) Cell viability was assessed using an MTT assay (*n* = 3). (**d**) Hoechst staining was used for nuclear visualization. (**f**) The apoptotic index is shown as the percentage of apoptotic cells relative to total cells, with counts performed in 10 fields (at least 30 cells per field). (**e**,**g**) Green fluorescence intensity was measured using DCFH-DA staining. (**h**) MMP was assessed by JC-1 dye staining. (**j**) The intensities of green and red fluorescence were measured using JC-1 staining. (**i,k**) The intensity of red fluorescence was measured using MitoSOX staining. *** *p* < 0.001 vs. negative control siRNA-transfected group. Arrow: apoptotic cells with nuclear DNA condensation. Si-NC, negative control siRNA-transfected; si-Nrf2, Nrf2 siRNA-transfected; Cont, negative control siRNA -transfected group; Dox, doxorubicin. SA, sinapic acid. Scale bar, 100 μm.

We further evaluated whether the protective effect of SA is mediated by Nrf2 signaling pathway in Dox-induced cardiotoxicity, the expression of Nrf2 protein was silenced by Nrf2 siRNA transfection. We demonstrated that Nrf2 siRNA effectively suppresses Nrf2 protein levels, evidenced by a remarkable reduction in Nrf2 protein levels in cells transfected with Nrf2 siRNA (Figure 8a,b). The viability assay revealed that cell viability was 50.0% in cells transfected with negative control (NC) siRNA and 39.9% in those transfected with Nrf2 siRNA, both treated with Dox- alone (Figure 8c). Especially, the cell survival rate of the Nrf2 siRNA-transfected cells with both 400 μM SA and Dox treatment was 53.9%, compared with that of NC-siRNA-transfected cells (Figure 8c). The analyses of Hoechst and DCFH-DA staining showed that apoptotic cells and ROS production were increased on 400 μM SA and Dox treatment in the Nrf2 siRNA-transfected cells with similar levels of those in NC-siRNA-transfected cells with Dox alone treatment (60.9% and 11.7-fold changes in apoptotic index and ROS production vs. NC-siRNA-transfected cells, respectively, Figure 8d–g). Finally, the analyses of JC-1 and MitoSox staining were found that MMP was also decreased and mitochondrial ROS production was increased with similar levels of those in NC-siRNA-transfected cells with Dox alone treatment (43.3% decrease and 65.9% of fluorescence intensities in JC-1 and MitoSox staining vs. NC-siRNA-transfected cells, respectively, Figure 8h–k). Overall, these findings showed that the protective effects of SA are abrogated when Nrf2 protein is knocked down, indicating that Nrf2 signaling mediates, at least in part, the protective effects of SA in Dox-treated H9c2 cardiomyoblasts.

## 4. Discussion

Dox is a widely used chemotherapeutic agent effective against various cancers, exerting its therapeutic effects by inducing oxidative stress, lipid peroxidation, membrane damage, mitochondrial dysfunction, and DNA damage [21,22]. However, the clinical use of Dox is limited due to its severe dose-dependent cardiotoxicity [23]. Therefore, our study sought to evaluate whether SA could protect against Dox-induced cardiac toxicity. SA is known for its potent antioxidant properties and potential benefits against various pathological conditions, including cancer, infections, anxiety, diabetes, neurodegeneration, and inflammation [11,24,25]. Previous research indicated that SA protects the heart from Dox-induced cardiotoxicity by inhibiting inflammation and apoptosis in rat models [18]. This study further investigated the mechanisms by which SA exerts its protective effects, focusing on its ability to mitigate oxidative stress and activate the Nrf2 signaling pathway, while also addressing pathological changes in Dox-treated cells.

Cell viability assays demonstrated that SA pretreatment significantly increased cell survival compared to Dox treatment alone, indicating that SA effectively rescues cells from Dox-induced cardiotoxicity. Apoptosis assays using nuclear staining revealed an increase in cells exhibiting nuclear condensation, a marker of apoptosis, in Dox-treated cells. In contrast, SA pretreatment reduced the number of apoptotic cells. Additionally, SA pretreatment modulated the apoptosis-related proteins, such as Bcl-2, Bax, and caspase 3, in a dose-dependent manner, further demonstrating its protective effects against apoptosis induced by Dox.

Oxidative stress plays a key role in Dox-induced cardiotoxicity [16,17]. Dox disrupts the endogenous antioxidant system and leads to ROS overproduction, which in turn causes oxidative stress and triggers intracellular pathological events such as DNA damage, lipid peroxidation, mitochondrial dysfunction, and apoptotic cell death [26]. Our findings align with previous studies showing that Dox induces ROS accumulation in cardiac cells [27,28,29]. Here, we observed that Dox treatment resulted in excessive ROS production and decreased levels of key endogenous antioxidants, including SOD1, GPx, and catalase. Importantly, SA pretreatment effectively inhibited ROS overproduction and preserved the levels of these antioxidants, suggesting that SA acts as a potent antioxidant against Dox-induced oxidative stress. Therefore, our findings suggest that SA has considerable potential against Dox-induced cardiotoxicity by mitigating oxidative stress and promoting antioxidant defenses.

Mitochondria play a central role in Dox-induced cardiotoxicity, primarily by disrupting mitochondrial function [30,31]. Given their abundance and critical role in meeting the high energy demands of the heart, mitochondria are particularly vulnerable to dysfunction [32]. Additionally, mitochondria are major sources of free radicals, contributing significantly to oxidative stress. The mitochondrial electron transport chain generates energy through ATP synthesis as electrons traverse the inner mitochondrial membrane [33]. During this process, electrons react with oxygen, leading to the production of superoxide or hydrogen peroxide, which are types of ROS. Under normal conditions, antioxidants such as SODs, catalase, and glutathione (GSH) effectively neutralize these free radicals [34]. However, when mitochondrial dysfunction occurs, as seen in pathological conditions, the antioxidant system’s ability to remove mitochondrial ROS is compromised [35]. Consistent with this, our study found that Dox treatment in H9c2 cardiomyoblasts induced mitochondrial dysfunction and oxidative stress due to increased mitochondrial ROS accumulation. Importantly, SA pretreatment significantly alleviated both mitochondrial dysfunction and ROS production.

ER stress is also linked to Dox-induced cardiotoxicity [36]. The ER is responsible for protein maturation functions, including folding, secretion, and post-translational modifications, which are crucial for maintaining normal protein function [37]. Pathological stimuli can impair ER functions, leading to ER stress characterized by the accumulation of unfolded or misfolded proteins. This stress can contribute to apoptotic cell death and exacerbate oxidative stress [38]. Our results indicate that Dox treatment activates several ER stress-related signaling proteins. Conversely, SA pretreatment effectively suppressed these changes, demonstrating its capacity to inhibit ER stress in Dox-treated H9c2 cardiomyoblasts. Taken together, the outcomes of our in vitro experiments revealed that SA effectively mitigates mitochondrial dysfunction and ER stress, both of which contribute to oxidative stress in Dox-induced cardiotoxicity.

The MAPK signaling pathway is crucial in mediating various cellular processes such as proliferation, differentiation, and migration [39]. It also plays a significant role in pathological conditions including inflammation, apoptosis, and oxidative stress, contributing to diseases such as cancer, Parkinson’s disease, and cardiovascular disorders [40]. Previous studies have highlighted that MAPK signaling pathways were involved in Dox-induced cardiotoxicity through both in vivo and in vitro models. Specifically, Dox administration increases the levels of p38 and JNK MAPK proteins, which contribute to the activation of apoptotic signaling pathways and the progression of heart failure in Dox-treated rats [41]. In Dox-treated H9c2 cardiomyoblasts, the MAPK signaling pathway has been linked to inflammation and cytotoxicity [42]. Recent research has identified that compounds or molecules that inhibit Dox-induced cardiotoxicity do so by suppressing the MAPK signaling pathway [43,44]. Our study confirmed that SA effectively inhibited the activation of typical MAPK proteins in Dox-treated cells. This indicates that SA protects H9c2 cardiomyoblasts from Dox-induced cardiotoxicity by modulating the MAPK signaling pathway.

We further investigated the Nrf2 signaling pathway to understand the protective mechanism of SA against Dox-induced cardiotoxicity. The Nrf2 protein has been extensively studied for its protective effects against disease, specifically by alleviating oxidative stress [45]. As a transcription factor, Nrf2 regulates the expression of antioxidative genes such as NQO1, HMOX1, and GCLC, thereby mitigating oxidative stress [46]. Evidence suggests that the Nrf2 signaling pathway is vital for maintaining cardiac function and protecting against various cardiac diseases [47]. Our results showed that Dox treatment led to the sequestration of Nrf2 in the cytosol and suppressed the expression of its target antioxidative genes. In contrast, SA pretreatment facilitated the translocation of Nrf2 to the nucleus and upregulated the expression of its target genes.

Finally, the knockdown study of Nrf2 was conducted to determine the cardioprotective effects of SA through Nrf2 signaling pathway. The positive effects of SA were diminished in Dox-treated cells that were silenced for Nrf2 following Nrf2 siRNA transfection, a marked decline in cell viability, an increase in apoptosis, an exacerbation of oxidative stress, and mitochondrial dysfunction, underscoring the critical role of Nrf2 in mediating the cardioprotective effects of SA. Overall, these demonstrate that SA protects H9c2 cardiomyoblasts from Dox-induced cytotoxicity by enhancing antioxidant defenses through modulation of the Nrf2 signaling pathway.

Indeed, previous research has indicated that SA protects the heart from Dox-induced cardiotoxicity by inhibiting inflammation and apoptosis, especially oxidative stress, in Dox- and streptozocin-treated rat models [18,19]. The current study provides an in-depth analysis of how SA exerts its protective effects, particularly focusing on reducing oxidative stress and its associated factors, such as mitochondrial dysfunction and ER-stress. Additionally, SA has been proven to activate the Nrf2 signaling pathway and its downstream antioxidative genes, while also addressing pathological changes in Dox-treated cells.

The current study has some limitations that should be noted: this study used the H9c2 cardiomyoblasts to evaluate the effect of SA on Dox-induced cardiotoxicity. Therefore, future studies should assess the effects of SA using the primary cardiomyocytes derived from neonatal rats. Additionally, since this study was conducted in vitro with H9c2 cardiomyoblasts to evaluate the protective effects of SA, it will be beneficial to use doxorubicin-treated animal models in future research. This approach aims to address the limitations of the current results obtained from cardiomyoblasts.

## 5. Conclusions

This study demonstrates that SA effectively mitigates Dox-induced cardiotoxicity in H9c2 cardiomyoblasts. The protective effect of SA is attributed to its antioxidant properties, which are mediated through the suppression of mitochondrial dysfunction and ER stress, facilitated by the activation of the Nrf2 signaling pathway. Although these findings are promising, they are solely based on in vitro experiments. Therefore, further research using animal models is essential to fully elucidate the protective effects of SA against Dox-induced cardiotoxicity and to validate its potential therapeutic benefits.

## Data Availability

The data are contained within the article.

## Supplementary Materials

The following supporting information can be downloaded at: https://www.mdpi.com/article/10.3390/antiox14030337/s1, Table S1: List of antibodies; Table S2: Specific primer sequences for qRT-PCR.
