# Supplementary material for: Sinapic Acid Ameliorates Doxorubicin-Induced Cardiotoxicity in H9c2 Cardiomyoblasts by Inhibiting Oxidative Stress Through Activation of the Nrf2 Signaling Pathway
Source: Antioxidants (Basel). 2025 Mar 13;14(3):337. doi: 10.3390/antiox14030337 (PMC11939272; doi:10.3390/antiox14030337)
Supplement: Supplementary file 1 [file antioxidants-14-00337-s001.zip › antioxidants-3443955-supplementary.pdf]

Supplementary Table 1. List of antibodies

Antibody	Catalog number	Working dilution	Company
B-cell lymphoma 2 (Bcl-2)	sc-492	1:1,000	Santa Cruz Biotechnology
Bcl-2-associated X protein (Bax)	#2772	1:1,000	Cell signaling
pro-caspase 3	#9665	1:1,000	Cell Signaling
Cleaved caspase 3	#9664	1:1,000	Cell Signaling
superoxide dismutase 1 (SOD1)	sc-101523	1:1,000	Santa Cruz Biotechnology
catalase	#14097	1:1,000	Cell signaling
Glutathione peroxidase 1/2 (GPx1/2)	#1341	1:1,000	Cell signaling
mitochondrial complex II protein	MS204	1:1,000	Abcam
phosphorylated pancreatic ER kinase (p-PERK)	ab192591	1:1,000	Abcam
PERK	#3192	1:1,000	Cell signaling
phosphorylated eukaryotic translation initiation factor 2α (p-eIF2α)	#3597	1:1,000	Cell signaling
(eIF2α	#9722	1:1,000	Cell signaling
activating transcription factor 4 (ATF4)	#11815	1:1,000	Cell signaling
C/EBP homologous protein (CHOP)	#2895	1:1,000	Cell signaling
phosphorylated extracellular signal-regulated kinase 1/2 (ERK 1/2)	#9101	1:1,000	Cell signaling
ERK 1/2	#9102	1:1,000	Cell signaling
Phosphorylated c-Jun N-terminal kinase (p-JNK)	#3251	1:1,000	Cell signaling
JNK	#9252	1:1,000	Cell signaling
Phosphorylated p38 (p-p38)	#9211	1:1,000	Cell signaling
P38	#9212	1:1,000	Cell signaling
nuclear factor-erythroid-derived 2-like 2 (Nrf2)	sc-365949	1:1,000	Santa Cruz Biotechnology
Thioredoxin 1 (Trx 1)	Sc-13526	1:1,000	Santa Cruz Biotechnology
β-actin	sc-47778	1:1,000	Santa Cruz Biotechnology
Lamin B1	Sc-374015	1:1,000	Santa Cruz Biotechnology

Supplementary Table 2. Specific primer sequences for qRT-PCR

Genes	Accession No.	Primers	
PGC1α	NM_031347	forward	5′-ACCGTAAATCTGCGGGATGA-3′
		reverse	5′-AGTTTCATTCGACCTGCGTAAAGTA-3′
ATP8	NC_012920	forward	5′-TGCCACAACTAGACACATCCA-3′
		reverse	5′-TGTGGGGGTAATGAAAGAGG-3′
ERRα	NM_001008511	forward	5′-GGCAATGCACTGAACATCGAG-3′
		reverse	5′-TGCTCCACAGCCTCAGCAT-3′
NRF	NM_001100708	forward	5′-CACTCTGGCTGAAGCCACCTTAC-3′
		reverse	5′-TCACGGCTTTGCTGATGGTC-3′
PPARα	NM_013196	forward	5′-TGAGCCCGGATATTGTAGCTGA-3′
		reverse	5′-GCCGAATAGTTCGCCGAAAG-3′
TFAM	NM_031326	forward	5′-TGAAGCTTGTAAATCAGGCTTGGA-3′
		reverse	5′-GAGATCACTTCGCCCAACTTCAG-3′
NQO1	NM_017000	forward	5′-TGAGCCCGGATATTGTAGCTGA-3′
		reverse	5′-GCATACGTGTAGGCGAATCCTG-3′
HMOX1	NM_012580	forward	5′-ATTTGTCCGAGGCCTTGAA-3′
		reverse	5′-CCAGGGCCGTATAGATATGGTA-3′
GCLC	NM_012815.2	forward	5′-GTGGACACCCGATGCAGTA-3′
		reverse	5′-CTTGTAGTCAGGATGGTTTGCAATA-3′
18S rRNA	M11188	forward	5′-TGCGGAAGGATCATTAACGGA -3′
		reverse	5′-CAGCCACCCGAGATTGAGCA -3′

PGC1α; PPARG coactivator 1α, ERRα; estrogen related receptor α, NRF; nuclear respiratory factor, PPARα; peroxisome proliferator activated receptor α, TFAM; transcription factor A, NQO1; NAD(P)H dehydrogenase, quinone, HMOX1; heme oxygenase 1, GCLC; glutamate-cysteine ligase, catalytic subunit
